# Treatment and outcome of metastatic parathyroid carcinoma: A systematic review and pooled analysis of published cases

**DOI:** 10.3389/fonc.2022.997009

**Published:** 2022-09-26

**Authors:** Andrea Alberti, Davide Smussi, Manuel Zamparini, Antonella Turla, Lara Laini, Chiara Marchiselli, Salvatore Grisanti, Paolo Bossi, Alfredo Berruti

**Affiliations:** Medical Oncology Unit, Department of Medical and Surgical Specialties, Radiological Sciences, and Public Health, University of Brescia at the Azienda Socio Sanitaria Territoriale (ASST)-Spedali Civili, Brescia, Italy

**Keywords:** parathyroid carcinoma, pooled analysis, primary hyperparathyroidism, treatment strategy, prognostic factors

## Abstract

**Background:**

Parathyroid carcinoma (PC) is an extremely rare malignant tumor with an incidence of about 6 new cases per 10 million inhabitants per year. While several papers have been published on treatments and outcomes of PC patients with loco-regional disease, little is known about the prognosis, treatment strategies, and prognostic factors of patients with distant metastasis.

**Materials and methods:**

We performed a systematic review and a pooled analysis of histopathologically confirmed PC cases published in literature using the following keywords: “metastasis–metastatic–secondary nodes” AND “parathyroid carcinoma”. Original case reports and case series reporting metastatic parathyroid carcinoma were included. Data from 58 articles were extracted in a piloted form by five reviewers on a shared database.

**Results:**

Seventy-nine patients with metastatic PC were identified between 1898 and 2018. Ten (13%) patients had synchronous metastases, while metachronous metastases occurred in 43 (54%) patients. The remaining 26 patients developed metastatic disease concomitantly to local recurrence. Primary hyperparathyroidism guided the diagnosis of metastatic recurrence in 58 (73%) patients. Surgery was the main primary approach adopted, as it was performed in 43 (54%) patients. Twenty (25%) patients underwent systemic antineoplastic therapy, consisting of chemotherapy, immunotherapy, tyrosine kinase inhibitors, and hexestrol therapy. Bone resorption inhibitors had a limited efficacy in the long-term control of hypercalcemia. After a median follow-up of 37.5 months, 43 (55%) patients died, 22 (51%) due to the consequences of uncontrolled PHPT. The median overall survival was 36 months (range: 1–252). Surgery was associated with a better OS (HR 0.48, 95% CI 0.26–0.88), whereas bone metastases represented a negative prognostic factor (HR 2.7, 95% CI 1.4–5.2).

**Conclusion:**

Metastatic PC has a relatively poor prognosis. The main goals of treatment are to counteract tumor growth and control hypercalcemia. Surgery of metastases is the best approach to achieve rapid control of PHPT and longer survival. Target therapies and immunotherapy deserve to be extensively tested in metastatic PC and strategies to better control hypercalcemia should be implemented.

## Introduction

Parathyroid carcinoma (PC) is an extremely rare malignant tumor with an incidence in the US SEER registry of about 6 new cases per 10 million population per year (1). PC accounts for about 0.005% of all cancers, and its prevalence in patients with primary hyperparathyroidism (PHPT) varies between 0.5% and 5% (2–4). Unlike benign parathyroid tumors, which are more frequent in female patients, PCs have an equal distribution in both sexes with an age at diagnosis between 45 and 55 years (3). PCs generally occur sporadically or less frequently in the context of familiar forms, particularly in 15% of cases of hyperparathyroidism-jaw tumor syndrome (HPT-JT) (hyperparathyroidism associated with ossifying fibroid of the jaw, cysts, and renal tumors) and more rarely in multiple endocrine neoplasia type 1 (MEN1) or type 2A (MEN2A) (5–7).

Approximately 90% of PC patients have symptomatic primary hyperparathyroidism (PHPT) (7). The most frequent symptoms are osteitis fibrosa, nephrolithiasis, neurocognitive disorders, and cardiac arrhythmia, while pancreatitis is less frequent (8–10). Clinical features caused by tumor invasion (neck masses, dysphagia, and hoarseness) are lately observed mainly in non-functioning PC (2), which accounts for less than 10% of all parathyroid carcinomas and occurs in an older population (sixth to seventh decade of life) (11–13).

Due to the early onset of PHPT, PC is usually diagnosed as a local disease and the definitive diagnosis is based on pathological features of capsular invasion, vascular invasion, and mitotic activity (2, 14). Molecular studies on PC have identified three main molecular mutated pathways: CDC73, CCND1, and PI3K/AKT/mTOR (14–17). Dysregulation of these pathways profoundly alters the balance between cell proliferation and apoptosis, and ultimately leads to a competitive growth advantage, metastatic competence, angiogenesis, and resistance to therapy in cancers (14–17).

Surgery is the mainstay of PC management. The gold standard is en bloc resection of all involved tissues and should include at least the PC and the ipsilateral thyroid lobe; the role of the central lymph node dissection is still debated. Surgery must be performed with care to avoid spillage of tumor cells into the surgical field (18).

Survival of PC patients is reportedly heterogeneous (1, 13). The estimated median overall survival is 14.3 years (13), with a 5-year survival rate of 85% and a 10-year survival rate of 49 to 77% (1, 19). Early diagnosis and radical surgery are associated with a better prognosis, while advanced age and lymph node metastases are predictive of a worse outcome (20–22).

The estimated risk of recurrence is 50%–60% at 2–5 years, more frequently in the case of a non-radical surgical approach (1, 22, 23). One-third of patients develop metastases, mainly in lung, liver, or bone (13, 24). The detection of locoregional or distant metastases is based on ultrasound of the neck, CT, or MRI of the chest and abdomen. Among nuclear medicine techniques, limited data are available in the metastatic setting. A total body scan with 99mTc-sestaMIBI and 18-FDG PET/CT may be complementary to conventional imaging in the initial staging, especially in more aggressive and rapidly evolving forms (25, 26).

While several papers have been published on the outcome of patients with early-stage disease, little is known about the prognosis, treatments, and prognostic factors of patients with relapsed and metastatic disease.

It has been reported that morbidity and mortality associated with metastatic PC is in many cases due to PHPT and related complications rather than to tumor progression (20). However, the proportion of patients who die from disease progression and that of patients who die from PHPT are unknown, as well as the impact on patient outcome of debulking surgery, systemic therapies, radiotherapy, and treatments administered to control hypercalcemia.

We systematically reviewed all cases of metastatic PC described in the literature, with the aim to perform a pooled analysis to obtain information on clinicopathological features, treatment strategies, patient outcome, and prognostic factors. Based on the results obtained, we provided some suggestions on possible clinical approaches.

## Methods and materials

### Identification of eligible articles and collection

PRISMA checklist was used to present the results of this systematic review

To the extent of the best published literature on metastatic PC, a four-step search strategy was planned. First, we identified the following keywords and MeSH terms in PubMed: “metastasis–metastatic–secondary nodes” AND “parathyroid carcinoma”. Secondly, the terms were searched in PubMed. Third, based on our objective, case reports and case series reporting metastatic parathyroid carcinoma were included in this review. Fourth, references to the included articles were scrutinized for additional papers. The last search was performed on 15 December 2021. Only articles that described the clinical history and treatment of individual patients with metastatic parathyroid carcinoma were selected. All articles that did not describe treatments for metastatic disease or did not report outcome or survival were excluded. No restrictions were imposed on publication date or publication status and only English-language articles were selected. The eligibility assessment was performed in an independent and unblinded standardized manner by five reviewers. Disagreements among reviewers were resolved by consensus.

Data were extracted by five reviewers on a shared database with collection and coding rules for the variables explained. After the first data extraction from a sample of included studies, the consistency of the extracted data was assessed to ensure that the reviewers who extracted the data interpreted the forms and draft instructions well. Each reviewer completed data extraction from each article independently. The following data were reported: (1) general information of the paper (first author, journal—the year of publication); (2) patient-disease data at first diagnosis (year of diagnosis, gender, age at diagnosis, clinical presentation, calcium level at diagnosis, PTH level at diagnosis, local–regional–systemic staging assessments, size of T, N-M status, treatment, type of surgery, margin status at the pathological examination, and neck dissection); (3) recurrence data (disease-free interval, site of recurrence, and first treatment of recurrence); (4) metastasis data (interval between first diagnosis and systemic relapse, sites, number, clinical presentation, treatment, chemotherapy drugs, the best response to any systemic lines of therapy, and progression-free survival of any systemic line); (5) hypercalcemia data (signs and symptoms due to hypercalcemia and systemic management of hypercalcemia); and (6) follow-up data (overall survival, follow-up status, and cause of death). If a full paper could not be retrieved, information was extracted from the abstract.

**Figure 1:**
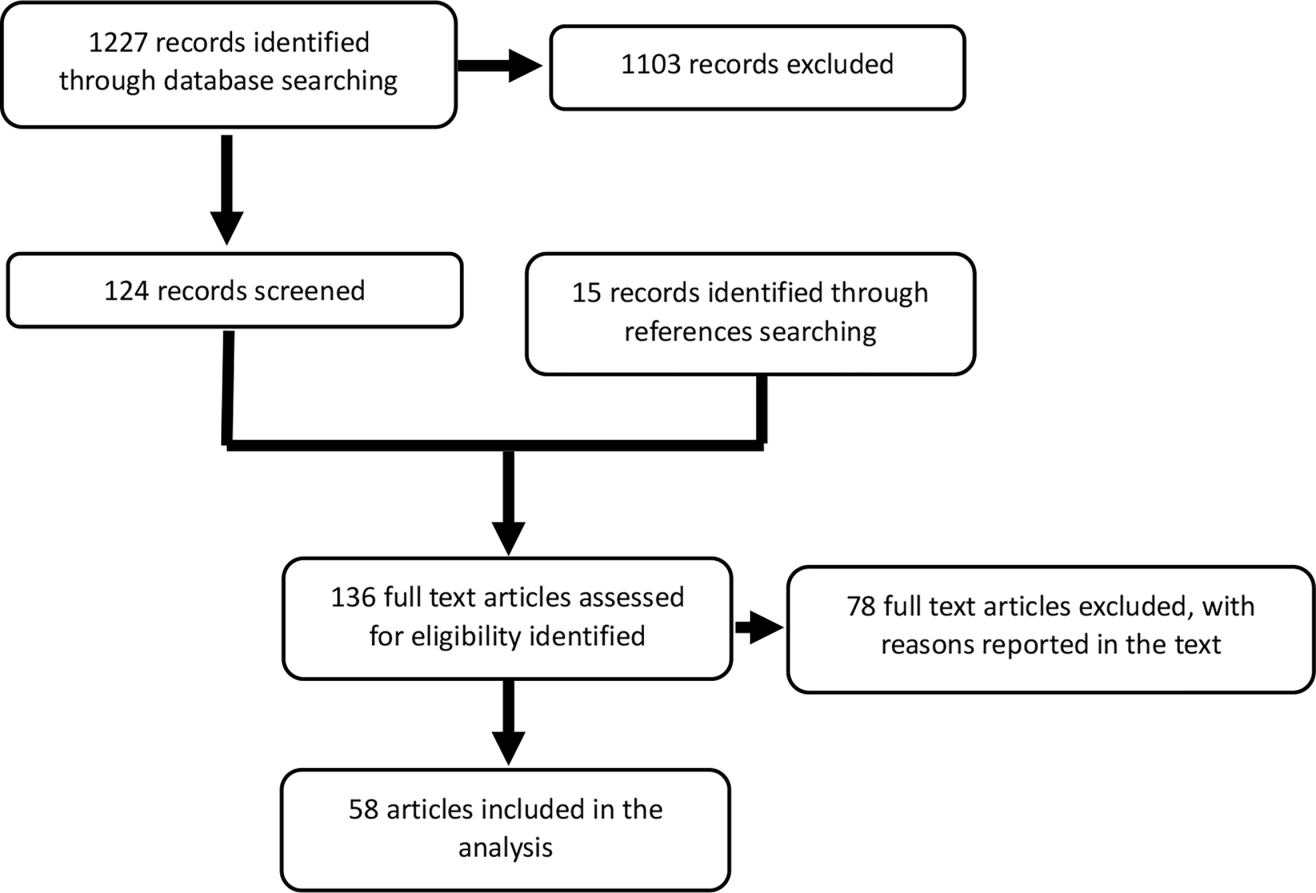
Consort diagram. The first search yielded 1,227 papers, 124 of which were relevant to this analysis. Among the references, we identified 15 additional articles. The absence of information on treatment or survival outcome excluded 78 articles. The final analysis included data from 58 articles.

The search revealed 1,225 potentially relevant articles on metastatic parathyroid carcinoma. Reading the title or abstract, 1,103 studies were excluded for various reasons (e.g., reviews–meta-analysis, no full-text available, *in vitro*, or animal). The full text of the remaining 122 articles was evaluated, and from the references of these studies, 15 additional articles were identified. Seventy-eight articles were excluded because they contained no data on treatment of metastatic disease, outcomes, or follow-up or because of possible duplicate use of data. Finally, data from 59 papers were included in this pooled analysis. **Figure 1** depicts the consort diagram.

### Statistical analysis

Descriptive statistics consisted of frequency tables of categorical variables and median (ranges) for continuous variables. Normal distribution of continuous variables was assessed by Kolmogorov–Smirnov and Shapiro–Wilk tests, when applicable. Cox proportional hazards models were introduced to investigate possible prognostic factors for overall survival. We accepted a type I error of maximum 5%. All analyses were performed with SPSS (IBM Corp. Released 2015. IBM SPSS Statistics for Windows, Version 23.0. Armonk, NY: IBM Corp.).

## Results

**Figure 2:**
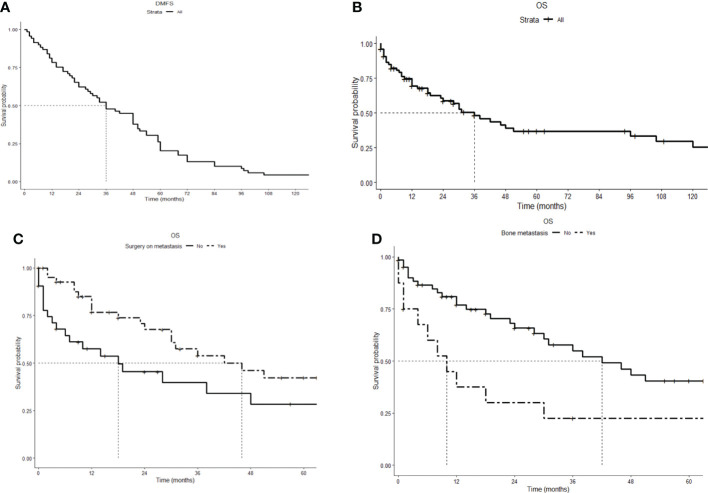
Kaplan–Meier survival curves. **(A)** Distant metastasis-free survival (DMFS): Time from the first diagnosis to diagnosis of metastasis, median 36 (range: 1–156) months. **(B)** Overall survival (OS): time from diagnosis of metastasis to death or last follow-up, median 36 (range: 1–252) months. **(C)** Overall survival comparison for cytoreductive surgery (HR 0.49, 95% CI 0.27–0.91). **(D)** Overall survival comparison for bone metastasis (HR 2.6, 95% CI 1.3–5.1).

We identified 79 patients diagnosed with metastatic PC between 1898 and 2018. Data of 59 of them (74%) were derived from case reports, 14 (18%) from single-institution case series, and 6 (8%) from multicenter series. **Table 1** depicts the characteristics of the patients. Forty-two patients (53%) were men and the median age at diagnosis was 45 years (range: 13–71). Five patients had an inherited susceptibility: HPT-JT syndrome in two patients, MEN1 in two patients, and neurofibromatosis 1 (NF1) in one patient. At first presentation, 10 patients (13%) had synchronous metastasis; the remaining 69 underwent surgery as the first approach on the primary tumor and the disease later relapsed with distant metastases ± local recurrence. In 26 patients (33%), metachronous metastasis occurred together with local relapse. The median time from the first diagnosis to metastasis diagnosis [distant metastasis-free survival (DMFS)] was 36 months (range: 1–156; **Figure 2A**). The most frequent site of metastasis was lung (57 patients, 72%), followed by bone (16 patients, 20%), liver (11 patients, 14%), extra-regional lymph nodes (8 patients, 10%), and brain (7 patients, 9%). At the first diagnosis of metastatic disease, 25 patients (32%) had more than 10 distant nodes, and even 30 patients (38%) had ≤4 nodes. Data on imaging techniques used for staging were heterogeneous and incomplete.

**Table 1:** Patient characteristics.

Patient features		No. (*N* = 79)
Age median (range)		45 (13–71)
Sex	Male	42 (53.2%)
PHPT at metastasis presentation		58 (73.4%)
Synchronous metastasis		10 (12.7%)
Metastasis site	Lung	57 (72.2%)
	Liver	11 (13.9%)
	Bone	16 (20.3%)
	Extraregional lymph nodes	8 (10.1%)
	Brain	7 (9%)
Number of distant nodes	1	14 (18.0%)
	2–9	26 (32.9%)
	≥10	25 (31.6%)
	Missing	14 (17.7%)
Signs and symptoms of PHPT	Renal lithiasis	32 (41.6%)
	Nervous symptoms	17 (22.1%)
	Pancreatitis	9 (11.7%)
	Osteitis	28 (36.4%)
	Arrhythmia	4 (5.2%)

### Strategies to control the tumor growth

Surgery was the primary approach for metastatic disease, as it was performed in 43 patients (54%). Thirty-five of them (81%) underwent resection of lung metastasis, while in six and four patients, surgery was performed on brain and liver metastases, respectively. In nine patients, surgery was followed by radiotherapy. No patients received adjuvant systemic therapy. Radiotherapy alone was the primary approach in 9 patients (12%); 20 patients (25%) underwent a systemic antineoplastic therapy, 11 (55%) of which as a primary approach (**Table 2**). The remaining 16 patients (20%) received only best supportive care. Systemic antineoplastic therapies consisted in chemotherapy in 10 patients and immunotherapy in 6, while tyrosine kinase inhibitors (TKIs) were prescribed in 5 patients and 2 patients received hexestrol therapy, a nonsteroidal estrogen. Fluorouracil + cyclophosphamide and dacarbazine (DTIC scheme) was the most used chemotherapy regimen. All four patients treated with DTIC achieved a clinical benefit from the therapy (i.e., disease response or stabilization) and median progression-free survival (PFS) was 10 months (range: 4–15 months). Two patients received anthracycline-containing schemes: methotrexate + cyclophosphamide + doxorubicin and lomustine (MAPP scheme) and doxorubicin alone, obtaining a partial response. Six patients treated with different monotherapies (vincristine, nitrogen mustard, paclitaxel, etoposide, cisplatin, capecitabine, and temozolamide) had no benefit from the treatment. Among the five patients treated with TKIs, none had a complete response, while a partial response was obtained in three of the four patients receiving sorafenib and in two patients receiving cabozantinib and regorafenib, respectively. It is noteworthy that regorafenib was administered as a second-line treatment in a patient already treated with sorafenib. Another patient received ramucirumab as a second-line approach, without any benefit. Among the patients receiving immunotherapy, four received an anti-human PTH immunotherapy with prolonged partial response in two patients (PFS of 144 and 32 months), stable disease for 6 months in the third, and PHPT control without tumor regression in the fourth. One patient received unspecified chemoimmunotherapy without benefit and one patient, with documented microsatellite instability, obtained a partial response lasting 24 months with pembrolizumab. **Table 2** summarizes the systemic treatments administered and the relative patient outcomes.

**Table 2:** Systemic therapies administered.

References	Drug	No. of patients	ORR	Median PFS (m) (range)	Median OS (m) (range)	Best outcome for hypercalcemia
(27, 28)	Estrogen	2	1/2	5.5 (1–10)	76 (72–80)	PR
(29)	Everolimus	1	0/1	2	Na	Na
(8, 30–32)	5FU + cyclophosphamide + dacarbazine	4	3/4	10 (4–15)	95 (35–162*)	CR
(8, 29, 33–35)	Other chemotherapies	6	0/6	2 (1–4)	83.5 (9–144)	PD
(8, 33, 34, 36)	Anthracycline monotherapy	4	1/4	3 (2–16)	23 (17–144)	PD**
(8)	Dacarbazine monotherapy	1	0/1	3	144	PD
(37)	Methotrexate + adriamycin + cyclophosphamide + lomustine	1	1/1	18*	29*	Na
(37, 38)	Immune checkpoint inhibitors	1	1/2	11.5 (2–21*)	14.5	CR
(29, 39)	Sorafenib	4	3/4	10 (3–17*)	22 (Na–22*)	CR
(15)	Cabozantinib	1	1/1	2–3	Na	Na
(15)	Ramucirumab	1	0/1	1	Na	Na
(15)	Regorafenib	1	1/1	2	Na	Na
(40–43)	Anti-PTH vaccine	4	2/4	NR (5–144*)	NR (10–144*)	CR

CR, complete resolution; Na, not assessed; NR, not reached; ORR, overall response rate; OS, overall survival; PD, progression disease; PFS, progression-free survival; PR, partial response.

* Censured.

** The patient, who benefited from chemotherapy, did not have PHPT.

° Other chemotherapies: vincristin, nitrogen mustard, paclitaxel, ethoposide, cisplatin, capecitabin, and temozolamid.

### Primary hyperparathyroidism: Features and outcomes

The diagnosis of metastatic disease was associated with PHPT in 58 patients (73%), while 10 (13%) patients reported symptoms caused by tumor invasion (e.g., neck masses, dysphagia, pain, and breathlessness) and 3 patients both. In the remaining eight patients (10%), the diagnosis was incidental during follow up. The median calcium level was 14.5 mg/dl (range: 9.1–22 mg/dl; 1st quartile [Q] 12.9 mg/dl, 2nd Q 14.5 mg/dl, 3rd Q 17 mg/dl, and 4th Q 22 mg/dl). As reported in **Table 1**, the most frequent symptoms associated with PHPT were, in descending order of frequency, renal lithiasis, nervous symptoms, pancreatitis, osteitis, and arrhythmia. Surgical treatment of metastases was effective in controlling calcium in all 33 patients for whom data were available (missing data for 10 patients). Twenty-five of them achieved normalization of serum calcium levels and the remaining eight patients had a reduction in calcium levels. Of these latter eight patients, five had a persistence of the disease after surgery. Data on the influence of radiotherapy on hypercalcemia were missing. Various systemic therapies were used for hypercalcemia: 24 patients (29%) received bone antiresorptive drugs, which consisted of bisphosphonates in 21 patients and denosumab in 3 patients. Eighteen of these patients (74%) achieved a benefit in controlling hypercalcemia, which was transient in 14 patients, while the duration of hypercalcemia control was missing in 4 patients. The remaining six patients had only transient stability of their calcium values for 2–6 months. Nine patients (12%) received calcitonin and six patients received other hormonal therapies (e.g., somatostatin analog). Two out of three patients had transient control of hypercalcemia after subcutaneous injection of octreotide. In five patients, hypercalcemia was accompanied by severe renal insufficiency requiring dialysis. Only 2 patients received cinacalcet. Due to incomplete records, data on the duration of hypercalcemia control were not analyzed. A logistic regression analysis was performed to test the association of the following variables with hypercalcemia: age, sex, lymph node status, metastasis at first presentation, sites of metastasis, surgery on metastasis, and radiotherapy; an inverse relationship was found with the presence of distant lymph nodes (HR 0.16; 95% CI 0.03–0.83).

### Survival analysis

After a median follow-up of 37.5 months, 43 patients (55%) died. The causes of death were as follows: uncontrolled PHPT in 22 patients (51%), tumor progression in 14 patients (32%), and other causes in 3 patients (7%). The cause of death was not specified for four patients. The median overall survival from diagnosis of metastasis to death or last follow-up (OS) was 36 months (range: 1–252; **Figure 2B**). As reported in **Table 3** and **Figures 2C, D**, cytoreductive surgery had an independent favorable role in predicting patients’ OS (HR 0.48, 95% CI 0.26–0.88), while bone metastases had a negative prognostic significance (HR 2.7, 95% CI 1.4–5.2). Information on individual patients can be found in the **Supplementary Material**.

**Table 3:** Prognostic factors of patients with metastatic parathyroid carcinoma according to univariate and multivariate analyses.

Overall survival	Univariate				Multivariate		
		HR	95% CI	*p*	HR	95% CI	*p*
Lung metastasis	Yes	0.483	0.239–0.976	0.043			
Liver metastasis	Yes	2.415	1.178–4.952	0.016			
Bone metastasis	Yes	2.704	1.395–5.243	0.003	2.303	1.138–4.663	0.020
Surgery on metastasis	Yes	0.478	0.260–0.878	0.017	0.474	0.246–0.912	0.025
Radiotherapy	Yes	2.184	1.131–4.220	0.020			
DMFS	>36 months	0.461	0.237–0.895	0.022			

## Discussion

Due to its extreme rarity, few data in the literature are available to guide the diagnosis and treatment of metastatic PC. The present pooled analysis of published cases confirms that PC occurs in patients in the fourth decade of age without difference between the two sexes. In most patients, the symptoms and signs leading to the diagnosis of distant relapse were related to PHPT, although 13% of patients reported symptoms caused by growth of the tumor mass (e.g., neck masses, dysphagia, pain, and breathlessness). These data have important implications on the follow-up of operated patients, which is based on prospective evaluation of blood levels of calcium and parathyroid hormone but should also include periodic radiological evaluations. Since, in the present series, the most frequent site of metastasis was lung, followed by bone, liver, and lymph nodes, a CT scan of the abdomen and thorax may be considered adequate in the follow-up. However, 9% of patients developed brain metastases; thus, a brain CT and/or MR should be considered in case of appearance of suspicious neurological symptoms. Noteworthy, in 26 patients (33%), distant metastases occurred together with local recurrence; this emphasizes the need to include accurate clinical examination and neck ultrasound in the follow-up. With regard to nuclear medicine techniques, the case series presented in literature suggest that a total body acquisition with 99mTc-sestaMIBI and 18F-FDG PET/CT can be complementary to conventional imaging in the initial staging, especially in more aggressive and rapidly evolving forms (25, 26). However, limited and heterogeneous data are available in the metastatic setting, since only few patients have been evaluated with these techniques. Therefore, we cannot draw any suggestions on the use of nuclear medicine techniques in the follow-up of PC patients after surgery. A frequent question about follow-up is its duration. In the present series, most metastatic recurrences occurred within 60 months; hence, follow-up should be continued for at least 5 years. It should be noted that one patient in this series developed metastases after 13 years, suggesting a possible follow-up extension beyond 5 years in selected cases.

The main goals of treating metastatic PC are to counteract tumor growth and control hypercalcemia. The control of hypercalcemia is of paramount importance, since in this series it was the cause of death in half of the patients. All PC patients with PHPT had at least one metastatic site; however, the extent of disease did not correlate with the severity of hypercalcemia. Therefore, surgery of metastasis with radical intent was effective in controlling hypercalcemia and should be pursued whenever possible, as it allowed rapid control of PHPT and a longer survival. This is relevant as many patients present an oligo metastatic disease at first relapse, and this observation, together with the predilection of the lung as a site of metastasis, favors surgery. If complete excision of all metastases is not possible, local regional treatments, such as radio-frequency ablation, similarly to what is adopted in other tumors, could reasonably be considered, although data on the efficacy of these treatments could not be obtained in this series.

With regard to systemic therapies, long-term control of hypercalcemia in malignancies can be achieved with the use of bone resorption inhibitors such as bisphosphonates and denosumab. In patients with PC-related hypercalcemia, however, the effect of these drugs was transient, and the syndrome was invariably resistant after initial control. Denosumab appears to be effective in patients with zoledronic acid resistance (44), but our case series does not allow us to obtain data in this regard, as only three patients were treated with denosumab. Good results have been obtained with calcimimetic drugs as cinacalcet, which is a recommended treatment (45, 46). However, no information on the effectiveness of this drug on patient outcome is available in this series. Two anecdotal case reports describe a response of hypercalcemia to estrogen therapy (27, 28). Furthermore, two patients had a transient improvement of PHPT in response to somatostatin analogue, but the results with this drug are still debated (47).

The prognosis of patients with metastatic PC was relatively poor, with a median survival of 36 months, but with considerable variability over a range of 1 to 255 months. The independent prognostic parameters were surgery as a favorable factor, underlining the importance of this therapeutic modality in this setting, and bone metastases as a worsening factor. This latter finding is in contrast with what has been observed in other metastatic malignacies (48, 49), in which the presence of visceral metastases plays a major role.

Data on the efficacy of systemic anticancer therapies are scarce and sometimes anecdotal. The few cases treated with chemotherapy have shown activity of alkylating drugs and anthracyclines. Target therapy could be a suitable option and several potentially actionable genomic alterations have been described, including PTEN, NF1, KDR, PIK3CA, and TSC242 (15, 16). In the present series, multikinase inhibitors targeting neoangiogenesis seem promising, as all drugs tested were active, although only five cases were considered. The same applies to immunotherapy, for which it is worth mentioning the interesting results obtained by a vaccine consisting of human and bovine PTH-like immunogenic fragments, which induced the formation of autoantibody against PTH and achieved lasting control of tumor growth and associated malignant hypercalcemia in three cases (40, 41, 50).Based on these very promising results, this treatment strategy deserves to be further tested either alone or in combination with modern checkpoint inhibitors.

In this study, we included the largest number of metastatic PC cases. Previous studies were mostly case reports or case series, and mostly included PC with and without recurrence. We focused on metastatic status, overall survival, and treatment, which were not highlighted in previous studies. To reduce potential bias in the analyses, we undertook a systematic review with the independent application of pre-defined inclusion criteria and data extraction. However, our study has some limitations. Demographics data were only derived from secondary data without standard protocols and from a long period; hence, there was high heterogeneity. Moreover, the study suffered from publication bias.

## Conclusion

Metastatic parathyroid carcinoma arises in the fourth decade of age and has a poor prognosis. Increased calcemia and parathormone values lead to diagnosis in most patients, although some report only symptoms caused by the growth of the tumor mass. Oligometastatic lung disease is the most frequent pattern of recurrence, and in about one-third of patients, local recurrence occurs together with distant metastases. The main goals of the treatment are to counteract tumor growth and control hypercalcemia. The latter challenge is of paramount importance, as it is the cause of death in at least half of patients. Surgery of metastases is the best approach to achieve rapid control of PHPT and longer survival. When surgery cannot be radical, other complementary local–regional approaches can be used. Systemic therapies should be considered when the disease could not be managed with local ablative treatments. According to the few data in the literature, dacarbazine, anthracyclines, sorafenib, and other antiangiogenetic drugs provided some positive results in the management of metastatic PC. Also, immunotherapy with vaccines based on bovine PTH-like immunogenic fragments seems promising, and this strategy deserves to be tested in association with modern immune checkpoint inhibitors. Based on the results of this systematic review and pooled analysis, we propose a list of suggestions for the management of patients with metastatic PC (**Table 4**).

**Table 4:** Suggestions for the clinical management of PC patients.

Follow-up of radically resected patients • After definitive treatment of the local disease, a patient follow-up should be implemented, including physical examination, blood calcium and parathyroid hormone levels, and periodic radiological imaging assessments. • Periodic radiological evaluations during follow-up should include neck ultrasound, and CT scan of the abdomen and thorax. Brain CT and/or MR should be considered in case of suspicious neurological symptoms. • Follow-up should be prosecuted for at least 5 years. • Total body 99mTc-sestaMIBI and/or 18F-FDG PET/CT scans can be complementary to conventional imaging in the initial staging.
Treatment • Surgery of metastasis should be persecuted as first approach whenever possible. • If complete removal of all metastases is not possible, other local treatments could reasonably be considered. • Systemic therapies should be considered in patients not amenable to surgery and/or local regional therapies.
Systemic treatment • Transient control of hypercalcemia can be achieved with the use of bone resorption inhibitors, such as bisphosphonates and denosumab. • Calcimimetic drugs, such as cinacalcet, are recommended treatment. • Dacarbazine and anthracyclines containing schemes are the chemotherapy of choice in the management of metastatic PC. • Anti-angiogenetic drugs and immunotherapy could also be possible options.

## Data availability statement

The original contributions presented in the study are included in the article/**Supplementary Material**. Further inquiries can be directed to the corresponding author.

## Author contributions

AA, AB, SG, and PB contributed to conception and design of the study. AA, DS, AT, LL, and CM contributed to the research and extraction of data. MZ performed the statistical analysis. AA, DS, AT, LL, and CM wrote the first draft of the manuscript. MZ wrote a section of the manuscript. All authors contributed to manuscript revision, read, and approved the submitted version

## Conflict of interest

The authors declare that the research was conducted in the absence of any commercial or financial relationships that could be construed as a potential conflict of interest.

## Publisher’s note

All claims expressed in this article are solely those of the authors and do not necessarily represent those of their affiliated organizations, or those of the publisher, the editors and the reviewers. Any product that may be evaluated in this article, or claim that may be made by its manufacturer, is not guaranteed or endorsed by the publisher.

## References

[1] Hundahl SA Fleming ID Fremgen AM Menck HR . Two hundred eighty-six cases of parathyroid carcinoma treated in the U.S. between 1985-1995: A national cancer data base report. Cancer (1999) 86:538–44. doi: 10.1002/(SICI)1097-0142(19990801)86:3<538::AID-CNCR25>3.0.CO;2-K 10430265

[2] Cetani F Pardi E Marcocci C . Update on parathyroid carcinoma. J Endocrinol Invest (2016) 39:595–606. doi: 10.1007/s40618-016-0447-3 27001435

[3] Talat N Schulte K-M . Clinical presentation, staging and long-term evolution of parathyroid cancer. Ann Surg Oncol (2010) 17:2156–74. doi: 10.1245/s10434-010-1003-6 20221704

[4] Salcuni AS Cetani F Guarnieri V Nicastro V Romagnoli E de Martino D . Parathyroid carcinoma. Best Pract Res Clin Endocrinol Metab (2018) 32:877–89. doi: 10.1016/j.beem.2018.11.002 30551989

[5] Givi B Shah JP . Parathyroid carcinoma. Clin Oncol (2010) 22:498–507. doi: 10.1016/j.clon.2010.04.007 PMC3781923 20510594

[6] Mohebati A Shaha A Shah J . Parathyroid carcinoma: challenges in diagnosis and treatment. Hematol Oncol Clin North Am (2012) 26:1221–38. doi: 10.1016/j.hoc.2012.08.009 23116578

[7] Al-Kurd A Mekel M Mazeh H . Parathyroid carcinoma. Surg Oncol (2014) 23:107–14. doi: 10.1016/j.suronc.2014.03.005 24742584

[8] Obara T Fujimoto Y . Diagnosis and treatment of patients with parathyroid carcinoma: An update and review. World J Surg (1991) 15:738–44. doi: 10.1007/BF01665308 1767540

[9] Levin KE Galante M Clark OH . Parathyroid carcinoma versus parathyroid adenoma in patients with profound hypercalcemia. Surgery (1987) 101:649–60. 3589961

[10] Motokura T Bloom T Kim HG Jüppner H Ruderman JV . A novel cyclin encoded by a bcl1-linked candidate oncogene. Nature (1991) 350:512–5. doi: 10.1038/350512a0 1826542

[11] Cetani F Frustaci G Torregrossa L Magno S Basolo F Campomori A . A nonfunctioning parathyroid carcinoma misdiagnosed as a follicular thyroid nodule. World J Surg Onc (2015) 13:270. doi: 10.1186/s12957-015-0672-9 PMC4563849 26350418

[12] Fingeret AL . Contemporary evaluation and management of parathyroid carcinoma. JCO Oncol Pract (2021) 17:17–21. doi: 10.1200/JOP.19.00540 32040373

[13] Harari A Waring A Fernandez-Ranvier G Hwang J Suh I Mitmaker E . Parathyroid carcinoma: A 43-year outcome and survival analysis. J Clin Endocrinol Metab (2011) 96:3679–86. doi: 10.1210/jc.2011-1571 21937626

[14] Porta C Paglino C Mosca A . Targeting PI3K/Akt/mTOR signaling in cancer. Front Oncol (2014) 4:64. doi: 10.3389/fonc.2014.00064 24782981 PMC3995050

[15] Kang H Pettinga D Schubert AD Ladenson PW Ball DW Chung JH . Genomic profiling of parathyroid carcinoma reveals genomic alterations suggesting benefit from therapy. Oncologist (2019) 24:791–7. doi: 10.1634/theoncologist.2018-0334 PMC6656481 30373905

[16] Pandya C Uzilov AV Bellizzi J Lau CY Moe AS Strahl M . Genomic profiling reveals mutational landscape in parathyroid carcinomas. JCI Insight (2017) 2(6):e92061. doi: 10.1172/jci.insight.92061 28352668 PMC5358487

[17] Hu Y Zhang X Wang O Bi Y Xing X Cui M . The genomic profile of parathyroid carcinoma based on whole-genome sequencing. Int J Cancer (2020) 147(9):2446–57. doi: 10.1002/ijc.33166 32574388

[18] Schulte KM Talat N Galata G Gilbert J Miell J Hofbauer LC . Oncologic resection achieving R0 margins improves disease-free survival in parathyroid cancer. Ann Surg Oncol (2014) 21:1891–7. doi: 10.1245/s10434-014-3530-z 24522991

[19] Busaidy NL Jimenez C Habra MA Schultz PN El-Naggar AK Clayman GL . Parathyroid carcinoma: A 22-year experience. Head Neck (2004) 26:716–26. doi: 10.1002/hed.20049 15287039

[20] Schaapveld M Jorna FH Aben KK Haak HR Plukker JT Links TP . Incidence and prognosis of parathyroid gland carcinoma: A population-based study in the Netherlands estimating the preoperative diagnosis. Am J Surg (2011) 202:590–7. doi: 10.1016/j.amjsurg.2010.09.025 21861982

[21] Sadler C Gow KW Beierle EA Doski JJ Langer M Nuchtern JG . Parathyroid carcinoma in more than 1,000 patients: A population-level analysis. Surgery (2014) 156:1622–30. doi: 10.1016/j.surg.2014.08.069 25456964

[22] Villar-del-Moral J Jiménez-García A Salvador-Egea P Martos-Martínez JM Nuño-Vázquez-Garza JM Serradilla-Martín M . Prognostic factors and staging systems in parathyroid cancer: a multicenter cohort study. Surgery (2014) 156:1132–44. doi: 10.1016/j.surg.2014.05.014 25444314

[23] Lee PK Jarosek SL Virnig BA Evasovich M Tuttle TM . Trends in the incidence and treatment of parathyroid cancer in the united states. Cancer (2007) 109:1736–41. doi: 10.1002/cncr.22599 17372919

[24] Wei CH Harari A . Parathyroid carcinoma: Update and guidelines for management. Curr Treat Options Oncol (2012) 13:11–23. doi: 10.1007/s11864-011-0171-3 22327883

[25] Evangelista L Sorgato N Torresan F Boschin IM Pennelli G Saladini G . FDG-PET/CT and parathyroid carcinoma: Review of literature and illustrative case series. World J Clin Oncol (2011) 2:348–54. doi: 10.5306/wjco.v2.i10.348 PMC3191327 22022662

[26] Hatzl M Röper-Kelmayr JC Fellner FA Gabriel M . 18F-fluorocholine, 18F-FDG, and 18F-fluoroethyl tyrosine PET/CT in parathyroid cancer. Clin Nucl Med (2017) 42:448–50. doi: 10.1097/RLU.0000000000001652 28394837

[27] Ellis JT Barr DP . Metastasizing carcinoma of the parathyroid gland with osteitis fibrosa cystica and extensive calcinosis. Am J Pathol (1951) 27:383–405. 19970978 PMC1937249

[28] Goepfert H Smart CR Rochlin DB . Metastatic parathyroid carcinoma and hormonal chemotherapy. Case report and response to hexestrol. Ann Surg (1966) 164:917–20. doi: 10.1097/00000658-196611000-00021 PMC1477091 5923121

[29] Bukowski RM Sheeler L Cunningham J Esselstyn C . Successful combination chemotherapy for metastatic parathyroid carcinoma. Arch Intern Med (1984) 144:399–400. doi: 10.1001/archinte.1984.00350140229032 6696578

[30] Calandra DB Chejfec G Foy BK Lawrence AM Paloyan E . Parathyroid carcinoma: biochemical and pathologic response to DTIC. Surgery (1984) 96:1132–7. 6505966

[31] Hakaim AG Esselstyn CB . Parathyroid carcinoma: 50-year experience at the Cleveland clinic foundation. Cleve Clin J Med (1993) 60:331–5. doi: 10.3949/ccjm.60.4.331 8339458

[32] Studentova H Melichar B Cincibuch J Kaminek M Frysak Z Geierova M . Brain metastases of parathyroid carcinoma: Review of the literature and a case report. BioMed Pap Med Fac Univ Palacky Olomouc Czech Repub (2015) 159:360–5. doi: 10.5507/bp.2015.001 25690525

[33] Kirkby-Bott J Lewis P Harmer CL Smellie WJB . One stage treatment of parathyroid cancer. Eur J Surg Oncol (2005) 31:78–83. doi: 10.1016/j.ejso.2004.06.014 15642430

[34] Barnes BA . Carcinoma of the parathyroid glands: Report of 10 cases with endocrine function. JAMA (1961) 178:556. doi: 10.1001/jama.1961.03040450020004 13865086

[35] Ordoñez NG Ibañez ML Samaan NA Hickey RC . Immunoperoxidase study of uncommon parathyroid tumors report of two cases of nonfunctioning parathyroid carcinoma and one intrathyroid parathyroid tumor-producing amyloid. Am J Surg Pathol (1983) 7:535–42. doi: 10.1097/00000478-198309000-00004 6353951

[36] Chahinian AP . Chemotherapy for metastatic parathyroid carcinoma. Arch Intern Med (1984) 144:1889. doi: 10.1001/archinte.1984.00350210219049 6477016

[37] Sturniolo G Gagliano E Tonante A Taranto F Papalia E Cascio R . Parathyroid carcinoma: Case report. G Chir (2013) 34(5–6):170–2. doi: 10.11138/gchir/2013.34.5.170 PMC3915582 23837957

[38] Park D Airi R Sherman M . Microsatellite instability driven metastatic parathyroid carcinoma managed with the anti-PD1 immunotherapy, pembrolizumab. BMJ Case Rep (2020) 13(9):e235293. doi: 10.1136/bcr-2020-235293 PMC7513558 32967944

[39] Rozhinskaya L Pigarova E Sabanova E Mamedova E Voronkova I Krupinova J . Diagnosis and treatment challenges of parathyroid carcinoma in a 27-year-old woman with multiple lung metastases. Endocrinol Diabetes Metab Case Rep (2017) 2017:16–0113. doi: 10.1530/EDM-16-0113 PMC5404464 28458892

[40] Sarquis M Marx SJ Beckers A Bradwell AR Simonds WF Bicalho MAC . Long-term remission of disseminated parathyroid cancer following immunotherapy. Endocrine (2020) 67:204–8. doi: 10.1007/s12020-019-02136-z PMC9361402 31782130

[41] Akirov A Asa SL Larouche V Mete O Sawka AM . The clinicopathological spectrum of parathyroid carcinoma. Front Endocrinol (2019) 10:731. doi: 10.3389/fendo.2019.00731 PMC6819433 31708875

[42] Horie I Ando T Inokuchi N Mihara Y Miura S Imaizumi M . First Japanese patient treated with parathyroid hormone peptide immunization for refractory hypercalcemia caused by metastatic parathyroid carcinoma. Endocr J (2010) 57(4):287–92. doi: 10.1507/endocrj.K09E-283 20051648

[43] Betea D Bradwell AR Harvey TC Mead GP Schmidt-Gayk H Ghaye B . Hormonal and biochemical normalization and tumor shrinkage induced by anti-parathyroid hormone immunotherapy in a patient with metastatic parathyroid carcinoma. J Clin Endocrinol Metab (2004) 89:3413–20. doi: 10.1210/jc.2003-031911 15240624

[44] Roukain A Alwan H Bongiovanni M Sykiotis GP Kopp PA . Denosumab for the treatment of hypercalcemia in a patient with parathyroid carcinoma: A case report. Front Endocrinol (2022) 12:794988. doi: 10.3389/fendo.2021.794988 PMC8842631 35173680

[45] Silverberg SJ Rubin MR Faiman C Peacock M Shoback DM Smallridge RC . Cinacalcet hydrochloride reduces the serum calcium concentration in inoperable parathyroid carcinoma. J Clin Endocrinol Metab (2007) 92:3803–8. doi: 10.1210/jc.2007-0585 17666472

[46] Takeuchi Y Takahashi S Miura D Katagiri M Nakashima N Ohishi H . Cinacalcet hydrochloride relieves hypercalcemia in Japanese patients with parathyroid cancer and intractable primary hyperparathyroidism. J Bone Miner Metab (2017) 35:616–22. doi: 10.1007/s00774-016-0797-0 27873072

[47] Denney AM Watts NB . The effect of octreotide on parathyroid carcinoma. J Clin Endocrinol Metab (2004) 89:1016–6. doi: 10.1210/jc.2003-031825 14764832

[48] Berruti A Libè R Laganà M Ettaieb H Sukkari MA Bertherat J . Morbidity and mortality of bone metastases in advanced adrenocortical carcinoma: a multicenter retrospective study. Eur J Endocrinol (2019) 180:311–20. doi: 10.1530/EJE-19-0026 30970324

[49] Largillier R Ferrero JM Doyen J Barriere J Namer M Mari V . Prognostic factors in 1,038 women with metastatic breast cancer. Ann Oncol (2008) 19(12):2012–9. doi: 10.1093/annonc/mdn424 PMC2733115 18641006

[50] Bradwell AR Harvey TC . Control of hypercalcaemia of parathyroid carcinoma by immunisation. Lancet (1999) 353:370–3. doi: 10.1016/S0140-6736(98)06469-1 9950443

