# Supplementary material for: Treatment and outcome of metastatic parathyroid carcinoma: A systematic review and pooled analysis of published cases
Source: Front Oncol. 2022 Sep 26;12:997009. doi: 10.3389/fonc.2022.997009 (PMC9550213; doi:10.3389/fonc.2022.997009)
Supplement: Supplementary file 1 [file DataSheet_1.pdf]

Reference	Year of diagnosis	Sex	Age at first diagnosis	PHPT (Y/N)	Synchronous metastasis (Y/N)	Disease free interval from first diagnosis to diagnosis of metastasis (m)	Calcium level at first diagnosis of metastasis (mg/dl)	Site of metastasis	Treatment of metastasis	OS (time from metastasis diagnosis to death)	Status	Cause of death
[1]	1996	M	62	Y	Y	_	17,23	Lung	RT and Systemic therapy	28	Alive	
[2]	2006	W	24	Y	Y	_	12,6	Lung	Surgery	144	Alive	
[3]	2008	M	71	Y	N	1	12,7	Liver	Radiofrequency ablation	9	Alive	
[4]	1949	W	53	Y	N	33	na	Liver	none	3	Died	Hypercalcemia related causes
[4]	1945	M	37	N	N	60	na	Lung, liver, bone and other	Systemic Therapy	0	Died	Disease progression
[4]	1947	W	31	Y	N	132	na	Lung	Surgery	12	Died	Hypercalcemia related causes
[4]	1961	W	53	na	N	36	na	Liver	none	0	Died	Hypercalcemia related causes
[5]	1992	W	50	Y	N	72	13,3	Lung	Surgery and Systemic therapy	32	Alive	
[6]	1975	M	54	Y	N	60	18	Lung	Systemic Therapy	15	Alive	
[7]	1982	W	33	Y	N	4	16,8	Other	Surgery and Systemic therapy	31	Died	Disease progression
[8]	1977	W	69	N	N	11	9,5	Lung + Extraregional lymph nodes	Surgery and Systemic therapy	18	Alive	
[9]	1955	W	56	N	N	14	13,7	Lung	none	19	Died	Hypercalcemia related causes
[10]	1996	M	35	Y	N	32	13,4	Extraregional Lymph nodes	none	1	Died	Disease progression
[11]	1998	W	63	Y	N	156	na	Bone	RT	0	Died	Disease progression
[11]	1998	M	50	Y	N	24	na	Lung	RT	192	Died	Other
[11]	1998	M	60	Y	N	12	na	Lung and Bone	RT	120	Died	Disease progression
[12]	1958	M	53	Y	N	22	11,5	Other	Surgery	1	Alive	
[13]	1945	W	26	Y	N	132	na	Liver	none	96	Died	Hypercalcemia related causes
[14]	1961	M	48	N	N	33	13,6	Lung	Surgery and Systemic therapy	51	Died	Hypercalcemia related causes

[15]	1967	M	58	Y	N	54	20,8	Bone and other	none	6	Died	Hypercalcemia related causes
[16]	1993	M	54	Y	N	60	16,2	Lung	Systemic Therapy	48	Died	na
[17]	2007	W	62	N	Y	_	9,5	Lung and Brain	Surgery and Radiotherapy and Systemic therapy	23	Died	Disease progression
[18]	1977	M	39	Y	N	84	15	Lung	none	7	Died	Hypercalcemia related causes
[19]	1970	W	38	Y	N	42	na	Lung	Surgery	30	Died	Hypercalcemia related causes
[19]	1977	W	43	N	N	11	na	Bone	Surgery	1	Alive	
[19]	1957	M	53	N	N	27	na	Liver and Bone	Surgery	106	Died	Hypercalcemia related causes
[20]	2001	W	54	Y	N	22	10,76	Liver, bone and brain	Surgery and RT	8	Died	na
[21]	2001	M	33	Y	N	2	15	Lung	none	27	Alive	
[22]	1990	M	44	Y	N	12	na	Lung and bone	Surgery and Systemic therapy	30	Died	Disease progression
[22]	1992	M	66	N	N	97	na	Lung and bone	RT	1	Died	Disease progression
[22]	2002	M	46	Y	N	6	na	Lung	Surgery and Systemic therapy	11	Alive	
[23]	1958	M	70	Y	Y	_	na	Other	Surgery	9	Alive	
[24]	1990	W	58	Y	N	30	13,2	Bone	Surgery	1	Alive	
[25]	1898	M	40	N	N	40		Bone	Surgery	36	Alive	
[26]	1971	W	21	na	N	36	na	Lung	Surgery and RT	12	Died	na
[27]	1958	M	61	Y	Y	_	na	Lung and liver	none	14	Died	Hypercalcemia related causes
[28]	1979	W	63	Y	N	51	18,04	Lung	Surgery	9	Died	Other
[29]	1953	W	53	Y	Y	_	na	Lung + Extraregional lymph nodes	none	252	Died	Other
[30]	1938	M	53	N	N	28	9,5	Skin	Surgery	1	Alive	
[31]	1987	W	40	Y	N	17	11,3	Lung	Surgery	98	Alive	
[32]	1971	W	71	Y	N	20	13.9	Lung, bone and skin	Surgery	4	Died	Hypercalcemia related causes
[33]	1979	M	45	Y	N	72	na	Lung	Surgery	28	Alive	
[34]	1972	W	13	Y	N	50	15,9	Lung	Surgery	202	Alive	
[34]	1979	M	37	Y	N	68	17,4	Lung	Surgery	94	Alive	
[34]	1975	M	23	Y	N	106	15	Lung	Systemic Therapy	38	Died	Hypercalcemia related causes

[34]	1987	M	64	Y	Y	_	15,6	Lung	Surgery	42	Died	Hypercalcemia related causes
[34]	1989	M	45	Y	Y	_	12,4	Lung and bone	Surgery and Radiotherapy and Systemic therapy	12	Died	Hypercalcemia related causes
[34]	1981	W	52	Y	N	99	12,5	Lung	none	57	Alive	
[34]	1971	W	34	Y	N	68	14,2	Lung	Surgery	196	Alive	
[35]	1975	M	27	N	N	19	9,2	Lung, Brain and Other	Surgery	8	Died	na
[35]	1977	M	59	N	N	4	9,5	Lung, bone and extraregional lymphnodes	Systemic Therapy	18	Died	Disease progression
[36]	1957	W	50	N	N	48	9,5	Skin	none	24	Alive	
[37]	2011	M	49	Y	N	10	14,4	Extraregional Lymph nodes	Surgery	2	Died	Disease progression
[38]	1953	W	20	Y	N	21	14.9	Lung	none	1	Died	Hypercalcemia related causes
[39]	2017	W	27	Y	N	17	16,8	Lung	Surgery and Systemic therapy	5	Alive	
[40]	2017	M	49	Y	Y	_	22,3	Lung and Brain	none	4	Alive	
[41]	1965	W	48	Y	N	48	18.0	Lung	Surgery	12	Alive	
[42]	1959	W	43	N	N	36	9,1	Pericardium	none	0	Alive	
[43]	1931	W	60	N	Y	_	na	Lung	none	4	Died	Disease progression
[43]	1930	M	49	N	N	7	na	Bone and Extraregional Lymphnodes	RT	1	Died	Disease progression
[44]	2005	M	35	Y	N	3	14,5	Lung	Systemic Therapy	2	Died	Hypercalcemia related causes
[45]	1981	W	34	Y	N	84	12,2	Lung	Surgery	60	Alive	
[46]	1995	W	20	Y	N	10	12,9	Lung	Surgery	2	Died	Disease progression
[47]	1994	M	44	Y	N	48	15,2	Brain	Surgery and RT	0	Alive	
[48]	1982	M	41	Y	N	59	18,84	Lung	Surgery	109	Alive	
[49]	1989	M	34	Y	N	24	16,03	Lung and Liver	Surgery	36	Died	Hypercalcemia related causes
[50]	1984	M	27	Y	N	14	13,03	Lung	Surgery and Systemic therapy	10	Alive	
[51]	1971	W	25	Y	N	50	18	Lung	Surgery and Radiotherapy	46	Died	Hypercalcemia related causes
[51]	1966	M	42	Y	N	48	17,5	Lung	Surgery and RT	24	Died	Hypercalcemia related causes

[52]	1949	M	46	Y	N	48	14,9	Lung	RT	28	Died	Hypercalcemia related causes
[53]	1985	W	45	Y	N	96	17,8	Lung, Brain and Other	Surgery	16	Alive	
[54]	2004	W	43	Y	N	72	13,2	Lung and Brain	Surgery	156	Alive	
[55]	1979	M	46	Y	N	29		Extraregional Lymph nodes	Surgery	1	Alive	
[55]	1975	M	21	Y	N	59	na	Lung and Extraregional Lymphnodes	Surgery	63	Alive	
[55]	1972	W	13	Y	N	60	17,2	Lung	Surgery and Systemic therapy	55	Alive	
[55]	1979	M	37	Y	N	59	na	Lung	Surgery	4	Alive	
[56]	2007	W	34	Y	N	2	14	Lung, liver and bone	Systemic Therapy	10	Died	Hypercalcemia related causes
[57]	2018	M	65	Y	N	8	11.5	Lung	Systemic therapy	24	Alive	
[58]	1945	W	29	Y	N	54	22	Lung and liver	Surgery	18	Died	Hypercalcemia related causes and disease progression

1. Bradwell, A. R. & Harvey, T. C. Control of hypercalcaemia of parathyroid carcinoma by immunisation. *Lancet* **353**, 370–373 (1999).
2. Sarquis, M. *et al.* Long-term remission of disseminated parathyroid cancer following immunotherapy. *Endocrine* **67**, 204–208 (2020).
3. Artinyan A, Guzman E, Maghami E, Al-Sayed M, D'Apuzzo M, Wagman L, Kim J. Metastatic parathyroid carcinoma to the liver treated with radiofrequency ablation and transcatheter arterial embolization. J Clin Oncol. 2008 Aug 20;26(24):4039-41. doi: 10.1200/JCO.2007.15.9038. PMID: 18711197.
4. Barnes, B. A. Carcinoma of the Parathyroid Glands: Report of 10 Cases with Endocrine Function. JAMA 178, 556 (1961).
5. Betea, D. et al. Hormonal and biochemical normalization and tumor shrinkage induced by anti-parathyroid hormone immunotherapy in a patient with metastatic parathyroid carcinoma. J Clin Endocrinol Metab 89, 3413–3420 (2004).
6. Bukowski, R. M., Sheeler, L., Cunningham, J. & Esselstyn, C. Successful combination chemotherapy for metastatic parathyroid carcinoma. Arch Intern Med 144, 399–400 (1984).
7. Calandra, D. B., Chejfec, G., Foy, B. K., Lawrence, A. M. & Paloyan, E. Parathyroid carcinoma: biochemical and pathologic response to DTIC. Surgery 96, 1132–1137 (1984).
8. Chahinian, A. P. Chemotherapy for metastatic parathyroid carcinoma. Arch Intern Med 144, 1889 (1984).
9. COOK PB. Hyperparathyroidism due to parathyroid carcinoma. J Bone Joint Surg Br. 1958 Aug;40-B(3):546-51. doi: 10.1302/0301-620X.40B3.546. PMID: 13575470.
10. Dionisi S, Minisola S, Pepe J, De Geronimo S, Paglia F, Memeo L, Fitzpatrick LA. Concurrent parathyroid adenomas and carcinoma in the setting of multiple endocrine neoplasia type 1: presentation as hypercalcemic crisis. Mayo Clin Proc. 2002 Aug;77(8):866-9. doi: 10.4065/77.8.866. PMID: 12173721.
11. Chow E, Tsang RW, Brierley JD, Filice S. Parathyroid carcinoma--the Princess Margaret Hospital experience. Int J Radiat Oncol Biol Phys. 1998 Jun 1;41(3):569-72. doi: 10.1016/s0360-3016(98)00098-4. PMID: 9635703.
12. EHRLICH A, McDONALD J, ZITANI A Jr. Carcinoma of the parathyroid with hyperparathyroidism and metastases. JAMA. 1961 May 6;176:453-5. doi: 10.1001/jama.1961.63040180013016d. PMID: 13726016.
13. Ellis, J. T. & Barr, D. P. Metastasizing Carcinoma of the Parathyroid Gland with Osteitis Fibrosa Cystica and Extensive Calcinosis. Am J Pathol 27, 383–405 (1951).
14. Goepfert, H., Smart, C. R. & Rochlin, D. B. Metastatic parathyroid carcinoma and hormonal chemotherapy. Case report and response to hexestrol. Ann Surg 164, 917–920 (1966).
15. Au WY. Calcitonin treatment of hypercalcemia due to parathyroid carcinoma. Synergistic effect of prednisone on long-term treatment of hypercalcemia. Arch Intern Med. 1975 Dec;135(12):1594-7. PMID: 128332.

16. Hakaim, A. G. & Esselstyn, C. B. Parathyroid carcinoma: 50-year experience at The Cleveland Clinic Foundation. Cleve Clin J Med 60, 331–335 (1993).
17. Studentova, H. et al. Brain metastases of parathyroid carcinoma: Review of the literature and a case report. Biomed Pap Med Fac Univ Palacky Olomouc Czech Repub 159, 360–365 (2015).
18. Ireland JP, Fleming SJ, Levison DA, Cattell WR, Baker LR. Parathyroid carcinoma associated with chronic renal failure and previous radiotherapy to the neck. J Clin Pathol. 1985 Oct;38(10):1114-8. doi: 10.1136/jcp.38.10.1114. PMID: 4056066; PMCID: PMC499451.
19. Aldinger KA, Hickey RC, Ibanez ML, Samaan NA. Parathyroid carcinoma: a clinical study of seven cases of functioning and two cases of nonfunctioning parathyroid cancer. Cancer. 1982 Jan 15;49(2):388-97. doi: 10.1002/1097-0142(19820115)49:2<388::aid-cncr2820490230>3.0.co;2-f. PMID: 7053835.
20. Kern M, Lee G, Robbins P, Bynevelt M, Watson P. Intracranial metastatic parathyroid carcinoma. Case report and review of the literature. J Neurosurg. 2004 Dec;101(6):1065-9. doi: 10.3171/jns.2004.101.6.1065. PMID: 15597772.
21. Khan MW, Worcester EM, Straus FH 2nd, Khan S, Staszak V, Kaplan EL. Parathyroid carcinoma in secondary and tertiary hyperparathyroidism. J Am Coll Surg. 2004 Aug;199(2):312-9. doi: 10.1016/j.jamcollsurg.2004.04.014. PMID: 15275889.
22. Kirkby-Bott J, Lewis P, Harmer CL, Smellie WJ. One stage treatment of parathyroid cancer. Eur J Surg Oncol. 2005 Feb;31(1):78-83. doi: 10.1016/j.ejso.2004.06.014. PMID: 15642430.
23. KLEINFELD G. A clinical and pathological study of 63 functioning parathyroid tumors. Cancer. 1959 Sep-Oct;12:902-11. doi: 10.1002/1097-0142(195909/10)12:5<902::aid-cncr2820120509>3.0.co;2-s. PMID: 14409734.
24. Koyano H, Shishiba Y, Shimizu T, Suzuki N, Nakazawa H, Tachibana S, Murata H, Furui S. Successful treatment by surgical removal of bone metastasis producing PTH: new approach to the management of metastatic parathyroid carcinoma. Intern Med. 1994 Nov;33(11):697-702. doi: 10.2169/internalmedicine.33.697. PMID: 7849385.
25. Langhans, T. Über die epithelialen Formen der malignen Struma. Virchows Arch. path Anat. 189, 69–152 (1907). https://doi.org/10.1007/BF02176714
26. Larmi TK, Kairaluoma MI, Huttunen R, Mokka R, Räsänen. Parathyroid carcinoma. A case report. Acta Chir Scand. 1976;142(6):471-4. PMID: 1007795.
27. LAZAR AW, LANDAU RL, KAPPAS A. Metastatic carcinoma of parathyroid and persistent hyperparathyroidism. Arch Pathol. 1961 Oct;72:484-9. PMID: 14463070.
28. Mann K. Oral biphosphonate therapy in metastatic parathyroid carcinoma. Lancet. 1985 Jan 12;1(8420):101-2. doi: 10.1016/s0140-6736(85)91989-0. PMID: 2856997.
29. McCance DR, Kenny BD, Sloan JM, Russell CF, Hadden DR. Parathyroid carcinoma: a review. J R Soc Med. 1987 Aug;80(8):505-9. doi: 10.1177/014107688708000816. PMID: 3309302; PMCID: PMC1290959.
30. McQuillan AS. Parathyroid tumor: report of two cases. Annals of Surgery, Sep 1938
31. Miki H, Sumitomo M, Inoue H, Kita S, Monden Y. Parathyroid carcinoma in patients with chronic renal failure on maintenance hemodialysis. Surgery. 1996 Nov;120(5):897-901. doi: 10.1016/s0039-6060(96)80101-2. PMID: 8909528.
32. Murray TM, Patt NL, Muzaffar SA. Parathyroid carcinoma: location of pelvic metastases by parathyroid hormone assay. Can Med Assoc J. 1974 Apr 6;110(7):809-10 passim. PMID: 4363399; PMCID: PMC1947441.
33. Obara T, Fujimoto Y, Tanaka R, Ito Y, Kodama T, Yashiro T, Kanaji Y, Yamashita T, Fukuuchi A. Mid-mediastinal parathyroid lesions: preoperative localization and surgical approach in two cases. Jpn J Surg. 1990 Jul;20(4):481-6. doi: 10.1007/BF02470837. PMID: 2201815.
34. Obara T, Okamoto T, Ito Y, Yamashita T, Kawano M, Nishi T, Tani M, Sato K, Demura H, Fujimoto Y. Surgical and medical management of patients with pulmonary metastasis from parathyroid carcinoma. Surgery. 1993 Dec;114(6):1040-8; discussion 1048-9. PMID: 8256207.
35. Ordoñez NG, Ibañez ML, Samaan NA, Hickey RC. Immunoperoxidase study of uncommon parathyroid tumors. Report of two cases of nonfunctioning parathyroid carcinoma and one intrathyroid parathyroid tumor-producing amyloid. Am J Surg Pathol. 1983 Sep;7(6):535-42. doi: 10.1097/00000478-198309000-00004. PMID: 6353951.
36. PACHTER MR, LATTES R. Uncommon mediastinal tumors. Report of two parathyroid adenomas, one nonfunctional parathyroid carcinoma and one "bronchial-type-adenoma". Dis Chest. 1963 May;43:519-28. PMID: 13940962.
37. Pyzik AJ, Matyjaszek-Matuszek B, Zwolak A, Chrapko B, Pyzik D, Strawa-Zakościelna K. Parathyroid cancer - difficult diagnosis - a case report. Nucl Med Rev Cent East Eur. 2016;19(1):46-50. doi: 10.5603/NMR.2016.0009. PMID: 26838944.
38. RAPOPORT A, SEPP AH, BROWN WH. Carcinoma of the parathyroid gland with pulmonary metastases and cardiac death. Am J Med. 1960 Mar;28:443-52. doi: 10.1016/0002-9343(60)90174-1. PMID: 14436266.
39. Rozhinskaya L, Pigarova E, Sabanova E, Mamedova E, Voronkova I, Krupinova J, Dzeranova L, Tiulpakov A, Gorbunova V, Orel N, Zalian A, Melnichenko G, Dedov I. Diagnosis and treatment challenges of parathyroid carcinoma in a 27-year-old woman with multiple lung metastases. Endocrinol Diabetes Metab Case Rep. 2017 Mar 13;2017:16-0113. doi: 10.1530/EDM-16-0113. PMID: 28458892; PMCID: PMC5404464.
40. Sadacharan D, Mahadevan S, Ferdinant J, Rakeshchandru K. Hypercalcaemic encephalopathy due to metastatic parathyroid carcinoma. BMJ Case Rep. 2017 May 31;2017:bcr2017219664. doi: 10.1136/bcr-2017-219664. PMID: 28566413; PMCID: PMC5747678.
41. Saunders KB, Richardson J. Carcinoma of the parathyroid. Proc R Soc Med. 1970 May;63(5):453-4. PMID: 5453422; PMCID: PMC1811821.
42. SIERACKI JC, HORN RC Jr. Nonfunctional carcinoma of the parathyroid. Cancer. 1960 May-Jun;13:502-6. doi: 10.1002/1097-0142(196005/06)13:3<502::aid-cncr2820130312>3.0.co;2-r. PMID: 14446529.

43. STEPHENSON HU Jr. Malignant tumors of the parathyroid glands; a review of the literature with report of a case. Arch Surg (1920). 1950 Feb;60(2):247-66, illust. doi: 10.1001/archsurg.1950.01250010265005. PMID: 15402807.
44. Sturniolo G, Gagliano E, Tonante A, Taranto F, Papalia E, Cascio R, Damiano C, Vermiglio F, Sturniolo G. Parathyroid carcinoma: case report. G Chir. 2013 May-Jun;34(5-6):170-2. doi: 10.11138/gchir/2013.34.5.170. PMID: 23837957; PMCID: PMC3915582.
45. Tominaga Y, Numano M, Uchida K, Sato K, Asano H, Haba T, Katayama A, Mukoyama A, Suzuki K, Tanaka Y, et al. Lung metastasis from parathyroid carcinoma causing recurrent renal hyperparathyroidism in a hemodialysis patient: report of a case. Surg Today. 1995;25(11):984-6. doi: 10.1007/BF00312388. PMID: 8640028.
46. Tseng CC, Huang JJ, Wang MC, Lan RR, Sung JM, Chen FF. Parathyroid carcinoma with multiple lung metastases. Nephrol Dial Transplant. 1999 Feb;14(2):449-51. doi: 10.1093/ndt/14.2.449. PMID: 10069211.
47. Tyler D 3rd, Mandybur G, Dhillon G, Fratkin J. Intracranial metastatic parathyroid carcinoma: case report. Neurosurgery. 2001 Apr;48(4):937-9; discussion 939-40. doi: 10.1097/00006123-200104000-00052. PMID: 11322456.
48. Vainas IG, Tsilikas C, Grecu A, Pasaitu K, Stergiou I, Kortsaris AH. Metastatic parathyroid carcinoma (mPCa): natural history and treatment of a case. J Exp Clin Cancer Res. 1997 Dec;16(4):429-32. PMID: 9505219.
49. van Haaren ER, Kievit J, Huysmans HA, van de Velde CJ. Successful resection of hepatic and pulmonary metastases in a patient with parathyroid carcinoma. Jpn J Clin Oncol. 1996 Apr;26(2):99-102. doi: 10.1093/oxfordjournals.jjco.a023192. PMID: 8609702.
50. Warrell RP Jr, Issacs M, Alcock NW, Bockman RS. Gallium nitrate for treatment of refractory hypercalcemia from parathyroid carcinoma. Ann Intern Med. 1987 Nov;107(5):683-6. doi: 10.7326/0003-4819-107-5-683. PMID: 2821862.
51. Flye MW, Brennan MF. Surgical resection of metastatic parathyroid carcinoma. Ann Surg. 1981 Apr;193(4):425-35. doi: 10.1097/00000658-198104000-00006. PMID: 7212805; PMCID: PMC1345095.
52. WEISSMAN I, WORDEN JP, CHRISTIE JM. Mediastinal parathyroid carcinoma with metastases; report of a case and review of the literature. Radiology. 1957 Mar;68(3):352-7. doi: 10.1148/68.3.352. PMID: 13420393.
53. Yamamoto T, Matsumura A, Fujita K, Kawakami Y, Yamashita K, Nose T. Cerebral metastasis of parathyroid carcinoma. Neurol Med Chir (Tokyo). 1996 Feb;36(2):96-8. doi: 10.2176/nmc.36.96. PMID: 8907011.
54. Yoshida S. Intracranial metastatic parathyroid carcinoma: case report. Surg Neurol. 2006 Jan;65(1):81-3. doi: 10.1016/j.surneu.2005.04.011. PMID: 16378868.
55. Fujimoto Y, Obara T, Ito Y, Kanazawa K, Aiyoshi Y, Nobori M. Surgical treatment of ten cases of parathyroid carcinoma: importance of an initial en bloc tumor resection. World J Surg. 1984 Jun;8(3):392-400. doi: 10.1007/BF01655086. PMID: 6464494.
56. Horie, I et al. First Japanese patient treated with parathyroid hormone peptide immunization for refractory hypercalcemia caused by metastatic parathyroid carcinoma. Endocr J. 2010
57. Park D, Airi R, Sherman M. Microsatellite instability driven metastatic parathyroid carcinoma managed with the anti-PD1 immunotherapy, pembrolizumab. BMJ Case Rep. 2020 Sep
58. Ellis HA, Floyd M, Herbert FK. Recurrent hyperparathyroidism due to parathyroid carcinoma. J Clin Pathol. 1971 Oct;24(7):596-604. doi: 10.1136/jcp.24.7.596. PMID: 4107487; PMCID: PMC477103.
